# High-throughput method for Oxygen Consumption Rate measurement (OCR) in plant mitochondria

**DOI:** 10.1186/s12870-023-04516-0

**Published:** 2023-10-17

**Authors:** Hanna Fuchs, Arleta Malecka, Adrianna Budzinska, Wieslawa Jarmuszkiewicz, Liliana Ciszewska, Aleksandra M. Staszak, Joanna Kijowska-Oberc, Ewelina Ratajczak

**Affiliations:** 1 Institute of Dendrology, Polish Academy of Sciences, Parkowa 5, Kornik, 62-035 Poland; 2 Department of Epidemiology and Cancer Prevention, Greater Poland Cancer Centre, Garbary 15, Poznan, 61-866 Poland; 3 Department of Bioenergetics, Faculty of Biology, Adam Mickiewicz University, Poznan, Uniwersytetu Poznanskiego 6, Poznan, 61-614 Poland; 4 Institute of Molecular Biology and Biotechnology, Faculty of Biology, Adam Mickiewicz University in Poznan, Uniwersytetu Poznanskiego 6, Poznan, 61-614 Poland; 5 Laboratory of Plant Physiology, Department of Plant Biology and Ecology Faculty of Biology, University of Bialystok, Ciolkowskiego 1J, Bialystok, 15-245 Poland

**Keywords:** Oxygen consumption rate, OCR, High-throughput plant mitochondria respiration assay, Plant mitochondria

## Abstract

**Background:**

Conventional methods to measure oxygen consumption, such as Clark-type electrodes, have limitations such as requiring a large amount of starting material. Moreover, commercially available kits for high-throughput methods are usually optimized for animal cells and mitochondria. Here, we present a novel method to measure the oxygen consumption rate using a high-throughput assay in isolated mitochondria of European beech seeds. To perform the measurements, we adapted the Agilent Seahorse XF Cell Mito Stress Test Kit protocol for measurements on plant mitochondria.

**Results:**

The optimized protocol for OCR measurement of mitochondria isolated from beech seeds allowed the observation of storage period-dependent gradual decreases in non-phosphorylating respiration, phosphorylating respiration and maximal FCCP-stimulated respiration. The longer the seeds were stored, the greater the impairment of respiratory function.

**Conclusions:**

Thanks to this method it is possible to minimize the amount of plant material and conduct research to obtain information on the respiratory condition and activity of plant mitochondria, including the efficiency of oxidative phosphorylation and the maximum oxidative capacity of the respiratory chain. We demonstrated that the improved protocol is suitable for study of plant material.

## Background

Seeds, like any plant organs, are subject to aging, which leads to a decrease in their viability and finally to a loss of their crucial property, which is the ability to germinate [1, 2]. The results of our earlier research indicate that the loss of viability of European beech (*Fagus sylvatica* L.) seeds during long-term storage is due to increased levels of reactive oxygen species (ROS), the lowered activity of the antioxidant system, changes in protein and sugar metabolism [2], and – consequently – changes in redox regulation [3]. Mitochondria are important organelles that not only participate in energy production but also play an important role in redox state regulation and cell signaling [4]. These organelles are major sites of ROS generation, while an excess of ROS destructively affects the structure of mitochondria and leads to their abnormal functioning [5]. We think, as do some other authors, that the process of seed aging is induced mainly by ROS and is associated with mitochondria [6–8]. However, research on the cellular respiration of trees and other plant seeds has limitations. The size of embryonic axes is small and conventional methods to measure oxygen consumption, such as Clark-type electrodes, require a large amount of starting material that must be amenable to stirring and prolonged suspension.

Currently, there are no high-throughput test systems to study plant seed respiration. We opted for plate technology, such as the Seahorse XFp analyzers, which allows for small-sample analysis, and the short analysis time compared to the Clark-type oxygen electrode method guarantees a reduction in the deterioration of mitochondria or the cell suspension. To date, the Seahorse XFp analyzer has not been used to study plant mitochondria. The combination of fluorophore-based micro oxygen sensors with microtiter plates allows bioenergetic measurements in adherent cell monolayers [9]. These multiwell, plate-based methods significantly reduce the required sample material and allow for simultaneous measurements of multiple experimental conditions. This technology can be used to study mitochondrial dysfunction, oxidative stress, specific pathways responsible for senescence and thorough analysis of cellular metabolism, including cellular respiration and extracellular pH [10]. Therefore, we present an optimized method to adapt the use of commercial microplate assays of oxygen consumption with the Agilent Seahorse XFp Analyzer (Agilent Technologies, Inc., Santa Clara, California, USA) in isolated mitochondria from beech seeds.

## Results and discussion

In this paper, we present the first protocol for a Mito Stress Test using the Seahorse Analyzer for plant mitochondria. Previously, it was used only for animal cells and mitochondria [11–15]. It is worth noting that the application of Seahorse technology in plant research has so far been limited to plant tissue analysis [9]. To optimize the method for plant mitochondria, we modified the protocol provided by the manufacturer. Isolated and purified mitochondria in a Percoll gradient from European beech seed axes stored for 2, 5, 8, and 15 years were resuspended in a pH 7.2 mitochondrial assay solution (MAS). Respiration reagent supplies were also suspended in MAS pH 7.2.

**Fig. 1:**
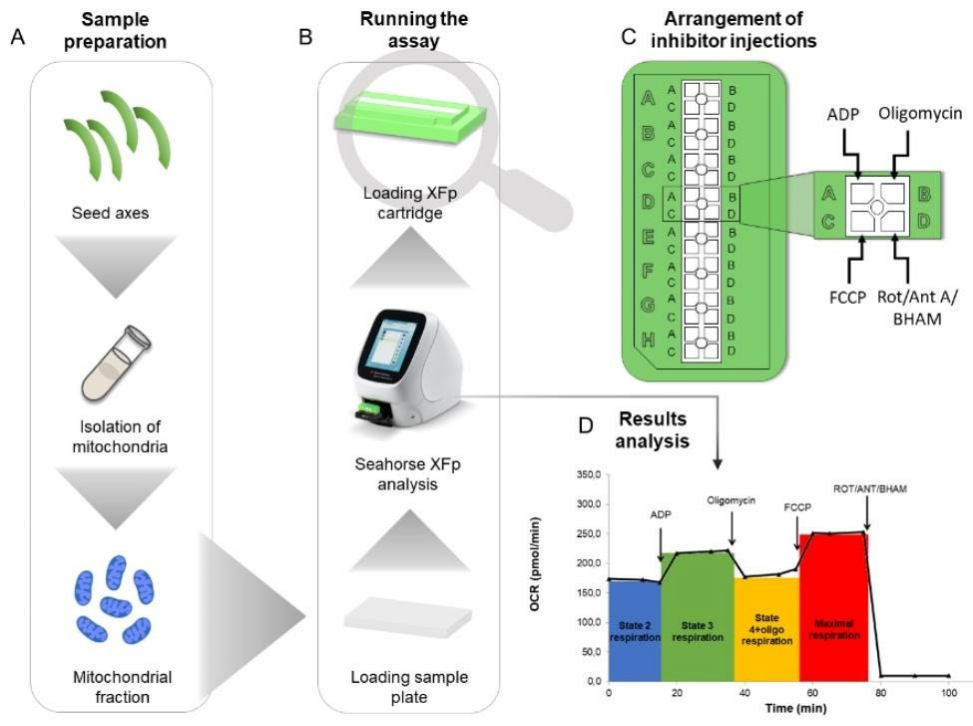
Plant mitochondria Mito Stress Test design and workflow. **A**) Sample preparation. Seed axes obtained from beech seeds stored for 2, 5, 8 and 15 years were subjected to the procedure of isolation of mitochondria by purification in a Percoll gradient. **B**) Running assay. Mitochondrial modulators placed onto the sensor cartridge and purified mitochondrial fractions loaded with mitochondrial assay solution on the wells in sample plate were inserted into the Seahorse XFp Analyzer, and then the assay was initiated. **C**) Arrangement of inhibitor injections. In order to maintain the established order of injections of the mitochondrial modulators into the sample wells, they were loaded in the appropriate ports in the cartridge. **D**) Results analysis. During the analysis injected inhibitors initiated the subsequent respiration states (ADP, Oligomycin, FCCP) or completely inhibited the oxygen consumption (Rot/Ant A/BHAM). Changes in the oxygen consumption rate (OCR) level occurring during the assay as a result of injection were recorded by Seahorse XFp Analyzer after every 3 min cycle of the assay and then the collected data were analyzed using Wave Software v. 2.6.1

Malate was used as respiratory substrate for complex I instead of the mixture of malate and pyruvate suggested in the original protocol. In this way, the activity of the respiratory chain involving the two main electron inputs (substrate dehydrogenases) can be measured. The protocol includes four injections instead of the original three: ADP, oligomycin, 4-(trifluoromethoxy)phenylhydrazone carbonyl cyanide (FCCP), and rotenone/antimycin A/benzhydroxamic acid (BHAM) (Fig. 1).

In the presence of malate, we measured State 2 respiration (basal respiration in the presence of respiratory substrate). First, ADP was injected to initiate State 3 respiration (phosphorylating respiration) for the duration of the measurement. Subsequently, oligomycin was injected to inhibit the activity of ATP synthase (complex V) and lead to State 4 respiration (nonphosphorylating respiration) with reduced activity of respiratory chain complexes due to ATP synthase not using the proton gradient. Then, the administered protonophore FCCP mimicked a physiological “energy demand” by stimulating the respiratory chain to operate at maximum capacity under uncoupling conditions. Finally, the administration of a mixture of three inhibitors, rotenone (an inhibitor of complex I), antimycin A (an inhibitor of complex III) and BHAM (an inhibitor of alternative oxidase present in the plant respiratory chain), led to complete inhibition of oxygen consumption by the tested plant mitochondria. The Seahorse XFp analyzer was operated in an air-conditioned room with a constant temperature of 19 °C, which allowed the analyses to be carried out at a constant temperature of 22 °C.

**Fig. 2:**
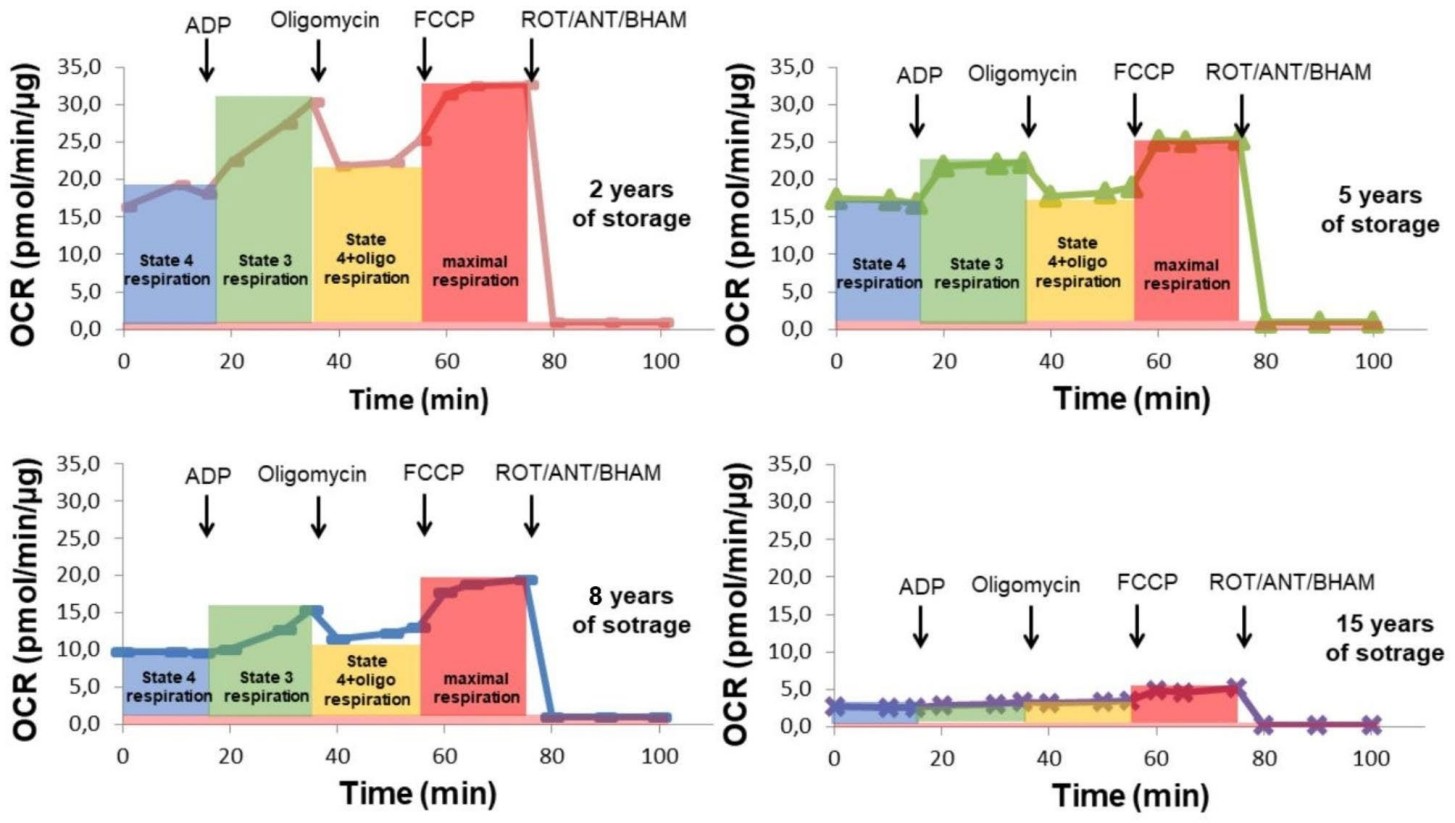
Oxygen consumption rate (OCR) was measured for mitochondria isolated from axes of seeds stored for 2, 5, 8 and 15 years. State 4 respiration, nonphosphorylating respiration in the presence of respiratory substrate without ADP; State 3 respiration, phosphorylating respiration in the presence of respiratory substrate and ADP; State 4_+ oligo_, nonphosphorylating respiration in the presence of respiratory substrate and oligomycin; maximal respiration, respiration in the presence of FCCP. OCR is presented in pmol O_2_/min/µg of mitochondrial protein

**Fig. 3:**
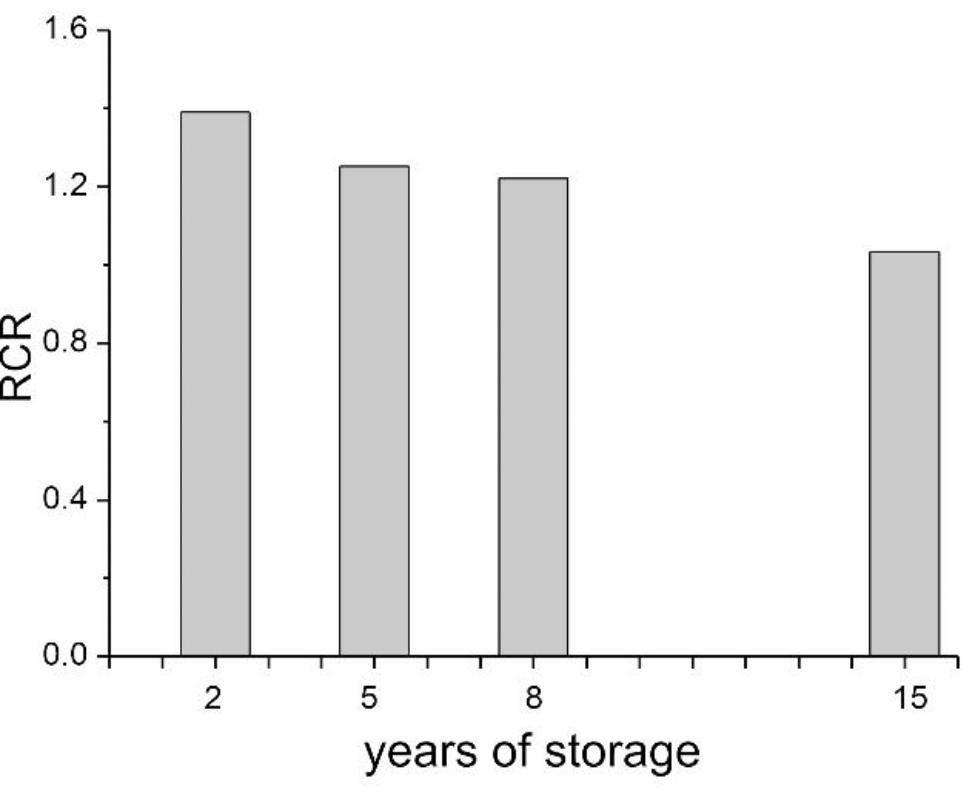
Respiratory control ratios (RCR, phosphorylating State 3 respiration versus nonphosphorylating State 4 respiration in the presence of oligomycin) calculated for mitochondria isolated from axes of seeds stored for 2, 5, 8 and 15 years

The OCR measurement protocol of mitochondria isolated from beech seeds allowed the observation of a storage period-dependent gradual decrease in nonphosphorylating respiration (in the absence or presence of oligomycin), phosphorylating respiration (ADP-stimulated State 3 respiration) and maximal FCCP-stimulated respiration (Fig. 2). The longer the seeds were stored, the greater the impairment of respiratory function. The highest respiratory activity (in all conditions) was observed in mitochondria from seeds stored for the shortest time, i.e., two years (Fig. 2). In the mitochondria from seeds stored for 15 years, we observed defective, dysfunctional and uncoupled mitochondria. As seed storage lengthened, mitochondria isolated from stored seeds had a decreasing respiration control ratio (RCR, ratio of ADP-stimulated respiration to nonphosphorylating respiration in the presence of oligomycin), indicating a weakening of the oxidative phosphorylation system and thus the efficiency of ATP synthesis (Fig. 3).

## Conclusions

Our preliminary research indicates that the Seahorse XFp Analyzer can be effectively used to study mitochondria isolated from plants, including tree seeds. Thanks to the Agilent protocol modified by us, it is possible to conduct research to obtain information on the respiratory condition and activity of mitochondria, including the efficiency of oxidative phosphorylation and the maximum oxidative capacity of the respiratory chain. This information is necessary for the analysis of the routes responsible for, among others, seed aging processes. We believe this could be a significant advance in the study of aging processes. Thanks to this method, we will be able to detect the signal of the seed aging process (decreased respiratory activity of mitochondria) accompanying the reduction in seed viability.

## Methods

### Plant material

Mature seeds of European beech were collected from a single tree growing in the Kornik Arboretum (Poland). Seeds were stored under controlled conditions specific for this species for 2, 5, 8, and 15 years. Axes of seeds were used to isolate mitochondria.

### Isolation of mitochondria

Seed axes (5 g, six biological replications) were homogenized in isolation buffer containing 5% bovine serum albumin (BSA), 1 mM ethylenediaminetetraacetic acid (EDTA), 1% polyvinylpolypyrrolidone (PVPP), 0.35 M sucrose, and 0.05 M KH_2_PO_4_/K_2_HPO_4_ buffer (pH 7.2). The homogenate was centrifuged for 10 min at 3,000 x g. Then, the supernatant was centrifuged for 20 min at 10,000 x g. The pellet was gently resuspended in a solution containing 0.3 M mannitol, 0.2% BSA, 1 mM EDTA, and 20 mM 3-(N-morpholino) propane sulfonic acid (MOPS) (pH 7.2) and then purified in a continuous gradient formed by 24% (v/v) Percoll in 0.25 M sucrose, 0.2% BSA and 20 mM MOPS (pH 7.2). The gradient was centrifuged at 40,000 x g for 30 min. Afterward, the mitochondrial fractions were carefully collected, washed to remove Percoll in a 20-fold volume of buffer (0.35 M sucrose, 20 mM MOPS, pH 7.2) and centrifuged three times for 30 min at 4,000 x g. The purified mitochondria were resuspended in 0.05 M KH_2_PO_4_/K_2_HPO_4_ (pH 7.2) with 0.35 M sucrose. The protein content was determined by adding up to 50 µL of 1 mL of mitochondria suspension 1:4 in Bradford reagent [16]. The absorbance was measured after 5 min on a spectrophotometer (Specord UV VIS) at 595 nm. The protein concentration was determined from the BSA standard curve.

### Determination of respiratory activity in mitochondria

The oxygen consumption rate (OCR), an indicator of mitochondrial respiration, was measured using the Mito Stress Test on an Agilent Seahorse XFp Analyzer (Agilent Technologies, Inc., Santa Clara, California, USA) according to the manufacturer’s instructions with modifications. The plate was prepared for calibration one day before the assay. Each well of the utility plate was filled with 200 µL of sterile water, and the moats around the outside of the wells were filled with 400 µL of sterile water per chamber. Afterward, the plate was incubated at room temperature overnight. The next day, approximately one hour before the assay, the water was replaced with XFp Calibrant at room temperature. A threefold concentrated mitochondrial assay solution pH 7.2 (3x MAS) (210 mM sucrose, 660 mM mannitol, 30 mM KH_2_PO_4_, 5mM MgCl_2_, 6 mM HEPES, 3 mM ethylene-bis(oxyethylenenitrilo)tetraacetic acid (EGTA), and 0,6% BSA) was prepared fresh on the day of assay. Respiration reagent stocks were resuspended in 3x MAS to final concentrations: 80 mM ADP, 30 µM oligomycin, 40 µM carbonyl cyanide-p-trifluoromethoxyphenyl-hydrazon (FCCP), 7.5 µM rotenone/7.5 µM antimycin A. Additionally, 15 mM benzhydroxamic acid (BHAM) was added to the rotenone/antimycin A solution. Mitochondrial modulators were loaded onto the sensor cartridge for final concentrations: port A, ADP (4mM); port B, oligomycin (1.5 µM); port C, FCCP (2 µM); and port D, rotenone/antimycin A/BHAM (0.5 µM/0.5 µM/1 mM, respectively). The mitochondrial suspension was diluted in 1x MAS containing 5 mM malate to a final concentration of 50 ng/mL mitochondrial proteins. Two hundred microliters per well (10 µg of mitochondrial protein) in three replications of mitochondrial suspension was seeded on XFp microplates for assessment of mitochondrial function and centrifuged for 20 min at 2.000 g, at 4 °C. After centrifugation, the supernatant was collected from the mitochondrial pellet and replaced by 200 µL 1x MAS with 5 mM malate.

The analysis was run using 3 × 3 min cycles with injection from sequential ports between each set. Data analysis was conducted using Wave Software v. 2.6.1 (Agilent Technologies, Inc., Santa Clara, California, USA).

## Data Availability

The datasets used and/or analyzed during the current study are available from the corresponding author on reasonable request.
